# Reported prevalence of childhood maltreatment among Chinese college students: A systematic review and meta-analysis

**DOI:** 10.1371/journal.pone.0205808

**Published:** 2018-10-15

**Authors:** Hanlin Fu, Tiejian Feng, Jiabi Qin, Tingting Wang, Xiaobing Wu, Yumao Cai, Lina Lan, Tubao Yang

**Affiliations:** 1 Department of Epidemiology and Health Statistics, XiangYa School of Public Health, Central South University, Changsha, Hunan Province, China; 2 Department of STD control and prevention, Shenzhen Center for Chronic Disease Control, Shenzhen, Guangdong Province, China; Georgia State University, UNITED STATES

## Abstract

**Objective:**

To estimate the prevalence of childhood maltreatment among college students in China by a systematic review and meta-analysis.

**Methods:**

A systematic search of relevant articles in Pubmed, Wanfang Data, Chinese Scientific Journals Fulltext Database (CQVIP), China National Knowledge Infrastructure (CNKI) and China Biology Medicine disc (CBMdisc) was conducted on September 1, 2017. A random-effects model was used to estimate the pooled prevalence and sources of heterogeneity were explored using subgroup analyses.

**Results:**

In total, 32 studies were included in our review. The pooled prevalence of childhood maltreatment among college students was 64.7% (CI: 52.3%-75.6%). For childhood physical abuse(CPA), childhood emotional abuse(CEA), childhood sexual abuse(CSA), childhood physical neglect(CPN)and childhood emotional neglect (CEN), the pooled estimates were 17.4% (13.8%-21.3%), 36.7%(25.1%-49.1%), 15.7%(11.6%-20.2%), 54.9%(41.2%-68.1%) and 60.0% (45.0%-74.0%), respectively. Use of the Childhood Trauma Questionnaire (CTQ) yielded a higher pooled estimate than any other measurement tools in the subgroup analyses of CPA, CEA, CSA, CPN and CEN. The Egger’s tests revealed no evidence of publication bias(*P*>0.05).

**Conclusions:**

Childhood maltreatment is common among college students in China. Prevention policies and programmes should be urgently developed to stop the occurrence of child maltreatment, and special attention should be paid to maltreated college students.

## Introduction

Childhood maltreatment, defined as the abuse and neglect of children under the age of 18, includes physical abuse, emotional abuse (also referred to as psychological abuse), sexual abuse and neglect[1]. Childhood maltreatment is a universal phenomenon. The past three decades have witnessed a mounting number of studies into the occurrence, prevalence and consequences of childhood maltreatment. According to the World Health Organization (WHO), over a quarter of adults worldwide reported being physically abused as a child, and 20% of women and 5–10% men reported being sexually abused in childhood[2]. A recent global meta-analytic study showed that, in pooled self-report studies, the estimated prevalence was 226/1000 for childhood physical abuse (CPA), 363/1000 for childhood emotional abuse (CEA), 127/1000 for childhood sexual abuse (CSA), 163/1,000 for childhood physical neglect (CPN) and 184/1,000 for childhood emotional neglect (CEN)[3–6].

Childhood maltreatment is a global public health problem with long-term consequences for an individual, their family and society. An abundance of evidence, mainly from retrospective studies and reviews, indicates that exposure to childhood maltreatment is associated with a range of adverse outcomes later in life[7–11]. For example, childhood maltreatment has been linked to depression, anxiety, drug and alcohol abuse, high-risk sexual behaviour, and even suicidal ideations and/or attempts[7–11]. Recently, in an Australian prospective birth cohort study, Abajobir and colleagues found that childhood maltreatment may predict cannabis use disorders[12], injecting drug use[13], risky sexual behaviours and pregnancy[14], high dietary fat intake[15], intimate partner violence victimization[16], lifetime delusional experiences[17] and asthma[18] in adulthood. Another longitudinal study also confirmed the association between childhood maltreatment and young adulthood alcohol, tobacco as well as cannabis use[19,20]. Moreover, increased severity of childhood maltreatment strongly correlated with adverse outcomes in adulthood [8].

In China, childhood maltreatment is also common. Although research in this field is still preliminary, many domestic studies have been published. Due to the lack of national epidemiological survey and national surveillance data on childhood maltreatment, estimates of the prevalence were based on reports of individual studies which varied widely[21]. In a meta-analysis of 10 studies, the combined prevalence of childhood maltreatment in China was 54% (95% CI: 42%~ 67%)[22]. Another systematic review covering all forms of childhood maltreatment showed that 26.6% of children in China suffered from physical abuse, 19.6% from emotional abuse, 8.7% from sexual abuse and 26.0% from neglect[23]. Furthermore, the economic burden as a result of the consequences of CPA, CEA and CSA were respectively calculated as 50 billion, 28 billion and 23 billion dollars[23]. Given that the article analysed the non-fatal health burden but ignored the mortality burden attributed to childhood maltreatment, the figure was clearly underestimated. Furthermore, Ji et al. [24,25]also performed two meta-analytic reviews of Chinese studies that focused solely on CSA and CPA, each result of which was different from that of the mentioned article above.

Despite several published reviews, to our knowledge, there is no systematic review on the prevalence of childhood maltreatment specifically for college students. College students are the mainstay of society who represent the future and hope of the motherland. College students with a developmental history of childhood maltreatment comprise an important but overlooked subgroup. As mentioned above, college students who are maltreated in childhood are at increased risk of developing high-risk behaviours and mental and physical diseases, and they are also likely to have poorer academic performance[10,11,26,27]. Effective interventions targeted at this subgroup should be taken to improve their current living conditions.

A reliable overall prevalence estimate of childhood maltreatment is crucial for health research, burden assessment, resource allocation and policy development. Consequently, we performed a meta-analysis to synthesize the heterogeneous results of previous studies on childhood maltreatment, covering CPA, CEA, CSA, CPN and CEN among college students in China. The overall goal was to provide a better and more accurate understanding of this subgroup, to draw the attention of family caregivers, educators and health researchers to this subgroup, and to promote child protection.

## Materials and methods

### Literature search

We conducted the present systematic review and meta-analysis strictly following the proposed PRISMA (Preferred Reporting Items for Systematic Reviews and Meta-analyses Protocols) statement. A systematic search of relevant articles in Pubmed, Wanfang, Chinese Scientific Journals Fulltext Database (CQVIP), China National Knowledge Infrastructure (CNKI) and China Biology Medicine disc (CBMdisc) was undertaken by two researchers on September 1,2017. The following search terms were used: “child” “childhood” “adolescent” “teenager” “youngster”, “maltreatment”, “abuse”, “neglect”, “trauma”, “violence”, “university”, “college”, “prevalence”, “incidence”, “rate” and “China”. An additional manual search of reference lists from systematic reviews or identified articles was performed to increase the number of relevant articles.

### Study selection

Eligible studies were included if they met the following criteria: 1) cross-sectional studies; 2) published in Chinese or English; 3) participants enrolled from Chinese college or university; 4) reported the maltreatment prior to 18 years old; 5) provided data that could calculate the prevalence of any form of childhood maltreatment (i.e., CPA, CEA, CSA, CPN and CEN). The exclusion criteria were as follows: 1) reported the scores, rather than percentage or no extractable data available; 2) conference abstract; 3) only described the prevalence of moderate-to-severe maltreatment, not all levels of maltreatment; 4) did not use a validated measurement to assess childhood maltreatment; 5) duplicate published articles or overlapping samples, and only studies providing detailed or maximum information retained.

In addition, to meta-analyse the prevalence of total childhood maltreatment, only studies that provided information on the prevalence of childhood maltreatment covering CPA, CEA, CSA, CPN and CEN were considered for inclusion.

### Data extraction and quality assessment

Using a study-designed standardized form, we extracted the available information from the included articles, such as first author, year of publication, geographic location, number of sampling sites, sampling method, measurement tool, sample size, response rate, number of maltreated, quality score and stratification variables (including gender and residence).

Methodological quality of the included study was evaluated based on a set of appraisal guidelines that was developed by Loney et al.[28]. The tool is structured with 3 broad organizing questions and contains 8 items: sampling method, sampling frame, sample size, standard measurement, outcome assessment, response rate with refusers described, confidence intervals and a description of subjects. Each item was assigned 1 point, and the total quality score of an article ranged from 0 to 8. A higher score indicated better the quality of the literature.

All the above work was separately performed by two researchers. Any discrepancy was resolved by consensus and was adjudicated by a third researcher if necessary.

### Statistical analysis

The pooled estimates of the prevalence of CPA, CEA, CSA, CPN, CEN and total maltreatment were performed using *R 3*.*1*.*2* software. Furthermore, 95% confidence intervals (CI) for the pooled prevalence were presented. In view of the possible heterogeneity underlying the included articles, we adopted results generated from random-effects model rather than fixed effects models based on between-study heterogeneity. Heterogeneity between studies was examined with Cochran’s chi-squared test (Cochran’s Q) and *I*^*2*^ values, with *P*<0.1 or *I*^*2*^>75% signifying considerable heterogeneity and *P* >0.1 or *I*^*2*^<50% signifying homogeneity. To identify the potential source of the heterogeneity, subgroup analysis was conducted with a random-effects model to compute the pooled estimates and corresponding 95% CIs according to the following grouping variables: number of sites (one college vs two or more colleges), sampling method (probability sampling vs non- probability sampling), measurement tool (Childhood Trauma Questionnaire (CTQ) vs Adverse Childhood Experience (ACE) vs Child Psychological Abuse and Neglect Scale (CPANS) vs Childhood Experience of Care and Abuse Questionnaire (CECA.Q) vs Personal Report of Childhood Abuse (PRCA)), sample size (<1000 vs ≥1000), response rate (<90.0% vs ≥90.0%), quality score (<5 vs ≥5), gender (male vs female) and residence (rural vs non-rural). In addition, by serially excluding each study from the analysis, we performed a sensitivity analysis to determine the robustness of the results. For the evaluation of publication bias, funnel plots and Egger’s linear regression analyses were performed. Unless otherwise specified, *P*<0.05 was defined as statistically significant for all tests.

## Results

### Literature retrieval and screening

**Fig 1:**
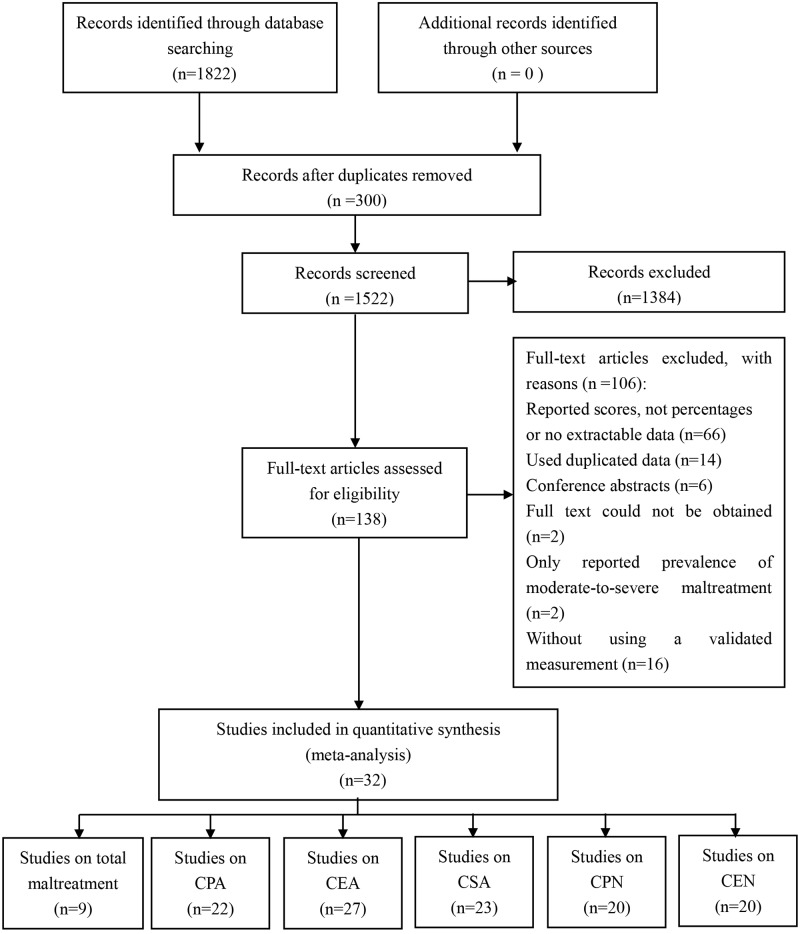
Flow chart of the selection of included studies for systematic review and meta-analysis.

In total, 1522 records were identified from the initial search. According to the inclusion and exclusion criteria, 32 studies were ultimately included for this meta-analysis (Fig 1), among which 9 studies[29–37] reported on total childhood maltreatment (6820 participants), 22 studies[30–51] reported on CPA (17164 participants), 27 studies [30–45,47–49,51–58]reported on CEA (19043 participants), 23 studies[30–45,47–51,59,60] reported on CSA (20276 participants), 20 studies[30–44,47–49,51,60] reported on CPN (16591 participants), and 20 studies [30–44,47–49,51,60]reported on CEN (16588 participants).

### Characteristics of the identified studies

The eligible studies were published from 2006 to 2017, with the sample size ranging from 213 to 2845. Five types of different validated measurement tools were adopted in the included studies, including CTQ (17 studies), CPANS (7 studies), ACE (5 studies), CECA.Q (2 studies) and PRCA (1 studies). One in three researchers conducted their surveys in two or more colleges, and a large majority of studies used probability sampling methods. The response rate ranged from 61.8% to 99.4% and the median value was 91.5%. As to the quality score, none of the articles met all eight criteria. The lowest and highest score were 3 and 7, respectively, with a median score of 6. Among them, 2 articles scored 3 points, 2 articles scored 4 points, 9 articles scored 5 points, 17 articles scored 6 points and 2 articles scored 7 points. The main problems were lack of random sample and sampling frame and not reporting confidence intervals for prevalence. Additionally, 10 articles provided information on stratification variables. An overview of the information about the studies can be found in Table 1.

**Table 1:** Characteristics of the included studies on the prevalence of childhood maltreatment among college students in China.

Study	Geographic location	Sampling method	Measurement tool	No. of sampling sites	Sample size	Response rate	No. of maltreatment						Quality score	Stratification variables
							Total	CPA	CEA	CSA	CPN	CEN		
Wang CP/2017	Shanxi	probability	CPANS	3	500	92.6%	NR	NR	227	NR	NR	NR	6	NR
Yang L/2017	Gansu, etc.	non-probability	CPANS	3	388	92.4%	NR	NR	102	NR	NR	NR	5	NR
Si Q/2017	Inner Mongolia	probability	CTQ	1	219	83.9%	NR	38	84	26	112	119	5	NR
Guo LY/2015	Liaoning	probability	CTQ	3	999	89.0%	NR	NR	NR	226	NR	NR	6	NR
Niu Y/2015	NR	probability	CTQ	1	2653	93.2%	NR	462	1719	599	1857	2134	6	NR
Li J/2015	Heilongjiang	probability	CTQ	4	929	91.6%	NR	174	584	155	748	763	5	gender, residence
Ma YJ/2015	NR	probability	CTQ	1	247	61.8%	NR	69	142	63	151	174	4	NR
Chen C/2015	Liaoning	probability	CTQ	1	809	89.9%	253	NR	NR	NR	NR	NR	6	NR
Jia GZ/2015	Shandong	probability	CTQ	4	1000	90.9%	NR	226	972	157	476	747	6	gender, residence
Guo LY/2015	Liaoning	probability	CTQ	1	217	90.4%	191	73	150	73	137	138	5	NR
Jin YY/2015	Anhui	probability	CTQ	1	932	94.6%	NR	106	325	179	200	375	6	gender, residence
Ji Y/2014	Hebei, etc.	non-probability	CTQ	>4	213	88.8%	NR	42	148	41	157	166	3	NR
Wang JH/2014	Heilongjiang	probability	CTQ	4	450	95.3%	212	33	181	54	189	186	6	gender, residence
Li WT/2014	NR	probability	CTQ	1	2845	92.9%	NR	560	NR	546	2096	2216	5	NR
Wang JH/2014	Heilongjiang	probability	CTQ	>4	475	95.0%	416	88	181	99	268	338	6	NR
Li HZ/2013	Zhejiang	NR	CPANS	2	468	93.6%	NR	NR	96	NR	NR	NR	4	NR
Cui NX/2013	Shandong	non-probability	ACE	1	492	91.5%	229	4	8	55	39	123	6	NR
Zhu XH/2012	Jiangsu	probability	PRCA	3	2374	97.6%	NR	337	745	38	NR	NR	6	NR
Ma JF/2012	Xinjiang	probability	ACE	1	475	99.4%	366	57	172	107	98	46	6	gender, residence
Fan YG/2011	Anhui	probability	ACE	1	1071	97.0%	728	288	41	94	149	287	6	gender, residence
Yuan H/2011	Tianjin	probability	CPANS	1	450	80.3%	NR	NR	97	NR	NR	NR	6	NR
Huang H/2011	Heilongjiang	probability	CPANS	2	448	89.6%	NR	NR	94	NR	NR	NR	6	NR
Yang SC/2011	Henan	probability	CECA.Q	1	733	97.7%	NR	34	NR	NR	NR	NR	6	NR
Ji Y/2011	Hebei	non-probability	CTQ	1	215	89.6%	NR	35	101	23	120	143	3	NR
Zeng Q/2011	NR	probability	CTQ	1	667	91.0%	NR	195	331	218	667	667	5	NR
He Y/2010	Hunan, etc.	non-probability	CTQ	3	412	96.0%	NR	111	266	110	371	368	5	NR
Su Y/2009	Anhui	probability	ACE	3	758	93.6%	454	237	27	17	217	126	7	gender
Xie ZJ/2008	Hunan	probability	CPANS	2	457	91.4%	NR	NR	99	NR	NR	NR	6	gender
Ling H/2008	Hunan	probability	CECA.Q	2	313	97.8%	NR	21	NR	21	NR	NR	6	NR
Cai XJ/2008	Inner Mongolia	probability	CTQ	1	270	90.0%	NR	47	122	54	230	227	5	NR
Liao Y/2006	Hunan	probability	CPANS	2	216	85.7%	NR	NR	45	NR	NR	NR	5	gender
Yao J/2006	Anhui	probability	ACE	3	2073	86.9%	1408	553	80	127	616	317	7	gender, residence

*Note*. NR = none reported; CPA = physical abuse; CEA = emotional abuse; CSA = sexual abuse; CPN = physical neglect; CEN = emotional neglect; CTQ = Childhood Trauma Questionnaire; CPANS = Child Psychological Abuse and Neglect Scale; ACE = Adverse Childhood Experience; CECA.Q = Childhood Experience of Care and Abuse Questionnaire; PRCA = Personal Report of Childhood Abuse

### Pooled prevalence of childhood maltreatment

**Fig 2:**
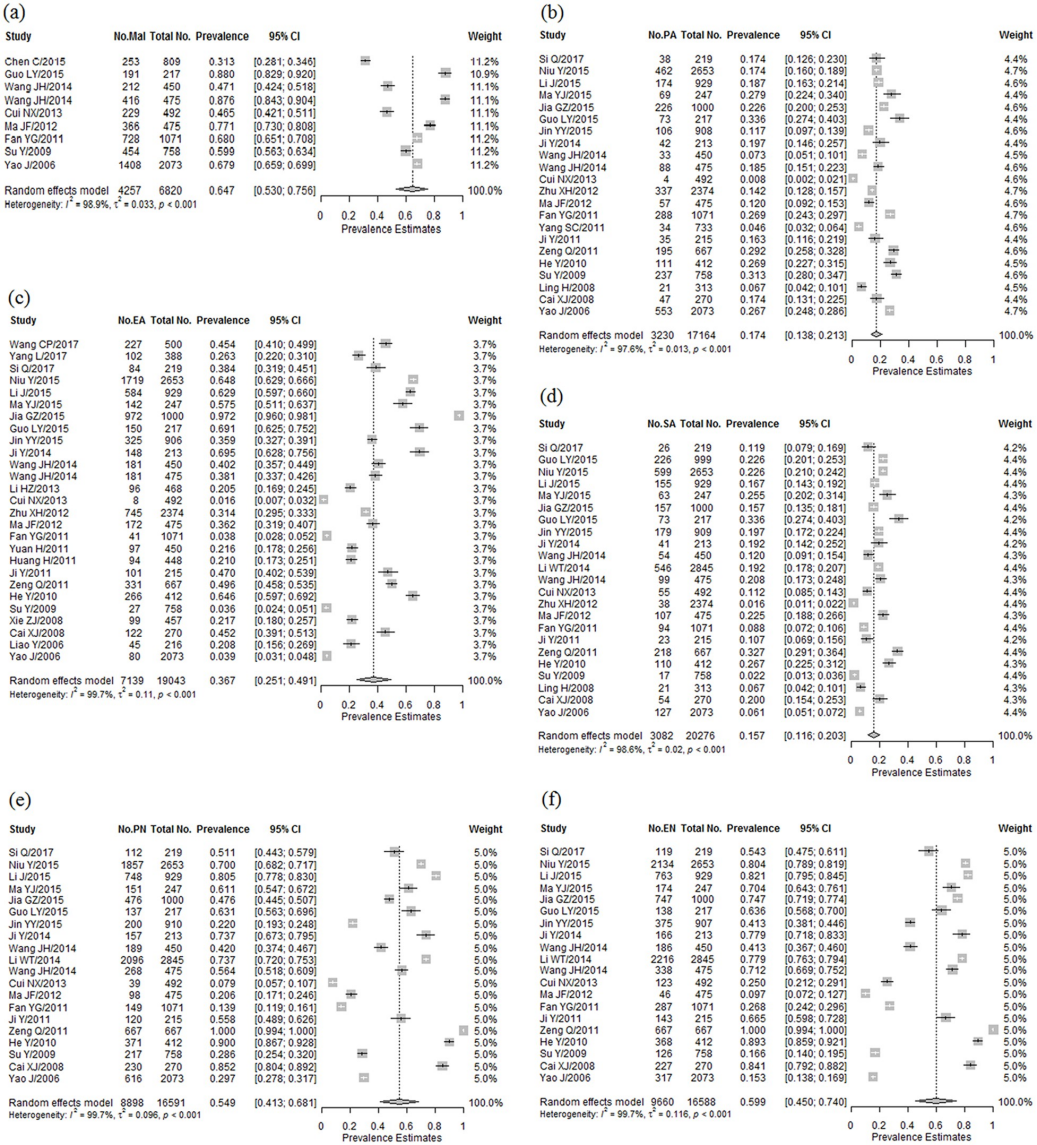
Forest plot of prevalence of total childhood maltreatment, CPA, CEA, CSA, CPN, and CEN among Chinese college students.

The point prevalence of total childhood maltreatment among college students reported by individual studies ranged from 31.3% to 88.0% (Fig 2a). Based on the 9 included studies, the pooled prevalence was 64.7% (CI: 52.3%-75.6%), with substantial heterogeneity (Q = 760.62, *I*^2^>95%, *p*<0.001). To explore the potential source of heterogeneity, we performed sub-group analyses. However, no significant differences between subgroups were found (Table 2).

**Table 2:** Subgroup meta-analyses of the prevalence of total maltreatment, CPA, CES, CSA, CPN and CEN by study characteristic.

Study characteristics	Prevalence,% (95% CI)					
	Total maltreatment	CPA	CEA	CSA	CPN	CEN
**Measurement tool**^a^						
ACE	65.5 [59.2; 71.6]	17.1 [7.5; 29.7]	7.2 [1.7; 16.1]	9.1 [4.5; 15.1]	19.5 [11.7; 28.6]	18.3 [12.9; 24.4]
CTQ	65.5 [33.9; 91.0]	19.8 [16.5; 23.3]	56.8 [44.8;68.4]	20.2 [17.8; 22.9]	67.2 [55.1; 78.2]	73.9 [64.7; 82.1]
PRCA	NA	14.2 [12.8; 15.6]	31.4 [29.5; 33.3]	1.6 [1.1; 2.2]	NA	NA
CECA.Q	NA	5.4 [3.9; 6.6]	NA	6.7 [4.2; 9.8]	NA	NA
CPANS	NA	NA	25.0 [18.6; 32.1]	NA	NA	NA
**Sampling method**						
Probability sampling	66.9 [54.4; 78.3]	18.3 [14.7; 22.1]	36.3 [23.6; 50.1]	15.6 [11.0; 20.7]	54.4 [39.8; 68.7]	58.4 [41.5; 74.3]
Non-probability sampling	53.5 [49.0; 57.9]	13.6 [2.1;32.5]	38.4 [10.8; 70.9]	16.5 [9.4; 25.1]	56.7 [13.5; 94.3]	66.1 [31.4; 93.0]
**Number of sites**						
One college	63.2 [43.0; 81.3]	16.4 [10.9; 22.7]	36.7 [21.0; 54.1]	19.2 [15.4; 23.4]	53.4 [33.5; 72.8]	60.5 [42.2; 77.3]
Two or more colleges	66.7 [52.3; 79.6]	18.6 [14.1; 23.6]	36.6 [20.1; 55.0]	12.2 [6.9; 18.8]	57.0 [39.5; 73.7]	59.1 [33.7; 82.2]
**Sample size**						
<1000	63.8 [46.4; 79.6]	16.3 [11.4; 21.8]	36.3 [27.1; 46.1]	17.6 [13.2; 22.4]	57.7 [38.7; 75.7]	61.6 [43.9; 77.8]
≥1000	67.9 [66.3; 69.6]	21.3[16.3;26.8]	38.1 [7.2;76.0]	11.1 [4.6;19.9]	46.4 [24.1; 69.4]	55.1 [25.9; 82.5]
**Response rate**						
<90.0%	49.6[16.2; 83.3]	21.0[16.7; 25.6]	34.3[17.3; 53.6]	15.9[9.1; 24.2]	60.0[38.6; 79.6]	61.6[30.3; 88.4]
≥90.0%	68.9[56.7; 79.9]	16.2[12.0; 20.9]	37.9[23.9; 53.0]	15.6[10.6; 21.4]	52.6[35.7; 69.3]	59.2[42.6; 74.8]
**Quality score**^a^						
<5	-^b^	21.2[14.8; 28.4]	48.2[25.2; 71.5]	18.1[10.3; 27.5]	63.7[53.1; 73.7]	71.7[65.0; 78.0]
≥5	64.7[53.0; 75.6]	16.8[13.0; 21.1]	34.7[22.3; 48.4]	15.4[11.0; 20.3]	53.3[38.3; 68.0]	57.8[41.2; 73.5]
**Gender**						
Male	72.6[60.5; 83.2]	26.7[23.5; 30.0]	25.4[10.8; 43.7]	12.7[4.1; 25.0]	45.6[28.0; 63.9]	39.4[19.0; 62.0]
Female	68.1[58.9; 76.7]	18.1[10.9; 26.6]	19.5[6.9; 36.3]	10.1[7.0; 13.8]	36.1[18.7; 55.6]	29.9[10.7; 53.8]
**Residence**						
Rural	71.3[62.3; 79.6]	21.0[16.9; 25.5]	22.2[1.0; 59.1]	9.9[3.3; 19.4]	56.5[31.9; 79.6]	47.5[14.9; 81.5]
Non-rural	68.9[56.6; 80.0]	27.1[19.1; 36.1]	22.5[2.5; 54.3]	10.8[4.6; 19.1]	41.6[13.7; 73.0]	43.6[9.3; 82.0]

Note:

^a^ There was significant difference for the variable.

^b^ None of the included articles had a quality score below 5.

CI = confidence interval; CTQ = Childhood Trauma Questionnaire; CPANS = Child Psychological Abuse and Neglect Scale; ACE = Adverse Childhood Experience; CECA.Q = Childhood Experience of Care and Abuse Questionnaire; PRCA = Personal Report of Childhood Abuse; CPA = childhood physical abuse; CEA = childhood emotional abuse; CSA = childhood sexual abuse; CPN = childhood physical neglect; CEN = childhood emotional neglect

### Pooled prevalence of CPA, CEA, CSA, CPN and CEN

Fig 2(b)–2(f) presents the prevalence for different forms of childhood maltreatment provided by a single study. The pooled estimates were 17.4% (13.8%-21.3%), 36.7% (25.1%-49.1%), 15.7% (11.6%-20.2%), 54.9% (41.2%-68.1%) and 60.0% (45.0%-74.0%) for CPA, CEA, CSA, CPN and CEN, respectively. Significant heterogeneity was observed for all subtypes of childhood maltreatment (*I*^2^>95%, *p*<0.001). Additionally, sub-group analysis was conducted to explain the heterogeneity. Although the variable measurement tool was related to heterogeneity, there were no significant between-group differences for any of the meta-analyses when studies were grouped by number of sites, sampling method, sampling size, response rate, quality score, gender or residence. The use of CTQ yielded a higher pooled estimate than any other measurement tools (Table 2).

### Publication bias and sensitivity analysis

**Fig 3:**
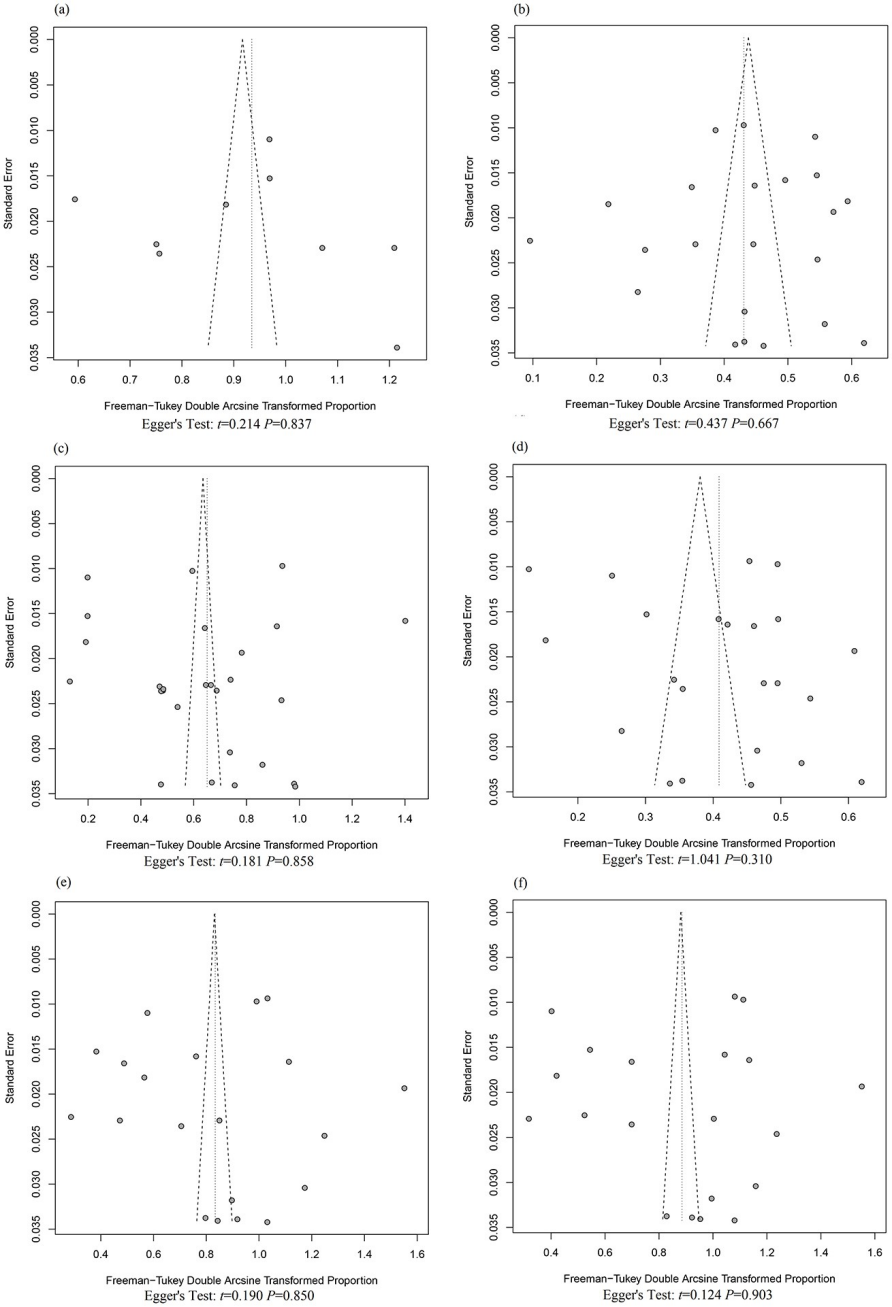
Funnel plots of the included studies with corresponding Egger’s test results. (a) Studies of total maltreatment. (b) Studies of childhood physical abuse. (c) Studies of childhood emotional abuse. (d) Studies of childhood sexual abuse. (e) Studies of childhood physical neglect. (f) Studies of childhood emotional neglect.

Sensitivity analyses were performed for the six meta-analyses. By serially excluding each study from the analyses, the pooled estimates varied slightly, indicating that the result was relatively stable. Furthermore, funnel plots revealed no asymmetry for each meta-analysis, and Egger’s tests showed that publication bias was unlikely (*P*>0.05) (Fig 3).

## Discussion

Based on a meta-analysis of 9 articles, the pooled prevalence of total childhood maltreatment was estimated at 64.7% (CI: 52.3%-75.6%), suggesting that childhood maltreatment is a widespread and serious problem among Chinses college students. The range of childhood maltreatment prevalence varied tremendously, from 31.3% to 88.0%. Compared with the previous meta-analysis conducted by Yang et al.[22], our prevalence estimate was slightly higher. We speculate that there are two main reasons for this observation. First, in Yang et al.[22], the subjects of the included studies consisted of children in addition to college students. Importantly, children under the age of 18 or even younger, have a relatively short exposure to maltreatment. Second, the selected studies for assessing childhood maltreatment in our article all used international validated measurements (i.e., CTQ & ACE) which covered at least five aspects, i.e., CPA, CEA, CSA, CPN and CEN. For Yang’s study[22], no threshold was established for this. Thus, comprehensive measurements might influence the result. Nevertheless, in spite of the geographical and cultural differences, our finding was roughly consistent with the U.S. results from surveillance data, the subjects of which were adults[61].

CPA is a topic of great concern. In the current analysis, the pooled prevalence of CPA was 17.4% (13.8%-21.3%), much lower than that reported in Fang’s and Ji’s findings[23,25]. Recall bias may partly explain this inconsistency. Compared with studies on children, studies on adults may be more likely to cause potential recall bias, leading to lower general estimate. As a consequence, when incorporating these studies in a meta-analysis, it can be a further source of bias[62]. In Fang’s and Ji’s studies[23,25], part of the included studies recruited children as subjects; in contrast, we only focused on studies on college students. On the other hand, we thought that disagreement between college students and children on the definition, attitude and cognition of CPA may play a role. Additionally, measurement tools can affect outcomes, as was indicated in our analysis.

CSA is another topic of many studies. Comparatively, CSA may leave a deep impression on individuals, especially penetrative CSA. The pooled prevalence of CSA in our study, 15.7% (11.6%-20.2%), appeared to be a slightly higher than the previous result estimated by Fang et al. of 8.7%[23], yet was consistent with the results of Ji’s and Peng’s meta-analysis[24,63]. As was pointed out by Andrews, sample type (i.e., college populations) is thought to be related to a higher prevalence of CSA[64]. Children who suffered CSA might have felt more ashamed than adults[65]; therefore, they may have been reluctant to disclose their victimization to researchers. Moreover, as we mentioned above, for children, the time-period for assessing CSA is limited. Based on the considerations above, we inferred that the prevalence of CSA from single studies that focused exclusively on college students, on the whole, might be higher than those that included children in their sample, as was the corresponding pooled estimates. However, this inference was not supported by a prior study[24]. Furthermore, measurement tools may have an impact on the results.

Despite lagging behind research on CPA and CSA, research on CEA is gradually receiving attention[4]. Substantiated evidence has suggested that CEA is a potential precursor for the development of psychological problems in adulthood[66,67]. The pooled estimate for the prevalence of CPA among college students was 36.7%(25.1%-49.1%), much higher than that for CPA and CSA. Furthermore, when integrating prevalence figures from studies using CTQ, the combined prevalence reached as high as 56.8%. Thus, CEA is common among college students.

Similar to CEA, childhood neglect has been overlooked in the research area of childhood maltreatment[6]. However, the adverse effects of neglect seem to be at least as damaging as those of abuse in the long term[7]. Although there are different subtypes of neglect, such as physical neglect, emotional neglect, educational neglect, and medical neglect, we focused on physical and emotional neglect, which were often involved in the evaluated studies. We found that the estimated prevalence was 54.9% (41.2%-68.1%) for physical neglect and 60.0% (45.0%-74.0%) for emotional neglect. It was obvious that the two figures were both fairly high. There was a significant difference between our result and Fang’s et al.[23], although it was not appropriate for enough direct comparison. In Fang’s meta-analysis[23], a total prevalence of neglect was presented (26.0%), regardless of subtype. In our view, the gap may also be attributable to lifetime exposure, measurement tools as well as the differential understanding of neglect. In the future, neglect should be given adequate attention.

In addition, the exploration of heterogeneity showed that, except for total childhood maltreatment, integrating prevalence from studies using CTQ presented higher combined rates in the sub-group analyses of CPA, CEA, CSA, CPN and CEN. Upon closer analysis, detailed items of each comparable dimension were observed in CTQ compared to ACE, CPANS, CECA.Q and PRCA. Even though the items of physical neglect and emotional neglect were the same in CTQ and ACE, the pooled prevalence of CPN and/or CEN provided by the two instruments were significantly different. Perhaps the discrepancy was related to differences in the sample, investigation method or investigators, although this remains uncertain. ACE also covered another dimension, for example, household dysfunction, which was not covered by CTQ. This may explain why there were no differences between the two instruments in assessing the total maltreatment prevalence. Moreover, subgroup analyses of the number of sites, sampling method, sampling size, response rate, quality score, gender and residence did not yield differences. However, it was noteworthy that, due to a lack of relevant literatures, the combined prevalence of different types of childhood maltreatment in different genders and residences may be both greater or less than the corresponding overall prevalence. Altogether, the sources of heterogeneity need to be further explored.

There were several limitations of this study. First, as mentioned above, after controlling for several moderator variables, the vast heterogeneity could not be resolved or fully interpreted. Therefore, our findings should be cited with caution. Second, all the included studies on childhood maltreatment among college students were cross-sectional; therefore, recall bias still cannot be excluded even after the integration.

Despite the above limitations, our findings have implications for practice, policy, and research. In view of the large differences among the measurement tools, a unified and better validated instrument needs to be considered for the future studies to provide better insights into childhood maltreatment. Importantly, based on these alarmingly high prevalence estimates from the current meta-analysis, it is evident that childhood maltreatment is quite common among college students in China. There is an urgent need for health researchers and policymakers to increase investment in evidence-based child maltreatment prevention by developing prevention policies and programmes, and child protection systems to stop the occurrence of child maltreatment. Family caregivers and educators should pay more attention to maltreated college students and use effective interventions and therapeutic strategies to help these students better adapt to the environment and society.

## Supporting information

S1 Checklist PRISMA Checklist. (DOC)

## Abbreviations

ACE: Adverse Childhood Experience
CBMdisc: China Biology Medicine disc
CEA: Childhood emotional abuse
CECA: Q: Childhood Experience of Care and Abuse Questionnaire
CEN: Childhood emotional neglect
CQVIP: Chinese Scientific Journals Fulltext Database
CI: confidence interval
CNKI: China National Knowledge Infrastructure
CPA: Childhood physical abuse
CPANS: Child Psychological Abuse and Neglect Scale
CPN: Childhood physical neglect
CSA: Childhood sexual abuse
CTQ: Childhood Trauma Questionnaire
PRCA: Personal Report of Childhood Abuse
